# Immune‐related lncRNA signature delineates an immune‐excluded subtype of liver cancer with unfavorable clinical outcomes

**DOI:** 10.1002/jcla.24244

**Published:** 2022-01-18

**Authors:** Yawei Chen, Leying Xi, Lihui Wei, Debin Sun, Tianmei Zeng

**Affiliations:** ^1^ Genecast Biotechnology Co., Ltd Wuxi China; ^2^ Department of Pediatrics Nanjing Hospital of Chinese Medicine Affiliated to Nanjing University of Chinese Medicine Nanjing China; ^3^ Department of Oncology Eastern Hepatobiliary Surgery Hospital Second Military Medical University Shanghai China

**Keywords:** immunotherapy, liver cancer, lncRNA, prognosis, tumor microenvironment

## Abstract

**Background:**

Long non‐coding RNAs (lncRNAs) play crucial roles in immune regulation and, therefore, may be closely related to the tumor microenvironment (TME). However, there are few studies regarding the relationship between the lncRNAs and the TME in liver cancer.

**Methods:**

Firstly, we constructed a lncRNA signature based on the top 10 immune‐inversely related lncRNAs obtained from the ImmLnc database and performed disease‐free survival (DFS) and overall survival (OS) analyses for the patients included in the Cancer Genome Atlas Liver Hepatocellular Carcinoma (TCGA‐LIHC) stratified by the lncRNA signature. Then, we explored the relationship between the lncRNA signature with distinct mutation profiles and the tumor microenvironment (TME).

**Results:**

The lncRNA signature was successfully constructed and verified by survival analysis. The high lncRNA signature was correlated with a decreased DFS and OS in liver cancer and other two gastrointestinal cancers. The mutation profiles showed that the Lnc_high group had a higher number of mutations on many genes, mostly enriched in p53 and WNT pathways. The TME results showed that the Lnc_high group had the highest proportion (51%) of lymphocyte depletion‐characterized immune subtype, and a higher expression of immune checkpoint molecules such as LAG3, PD‐L1, CTLA4. On the contrary, in the Lnc_low group, infiltrating immune‐cell proportions were significantly higher, and a significant enhancement of four axes of the cancer immunity cycle immunogram was observed in this group.

**Conclusions:**

The lncRNA signature we constructed identified an immune‐excluded subtype of liver cancer with unfavorable clinic outcomes, which could be tested as a biomarker for immunotherapy in the future.

## Graphical abstract

We constructed a novel immune‐related lncRNA signature based on lncRNAs that affect immune pathway activity and determined that this lncRNA signature correlated with the progression and prognosis of liver cancer. The lncRNA signature had a significant relationship with tumor mutations and the TME and may be integrated into a comprehensive biomarker system for liver cancer immunotherapy.

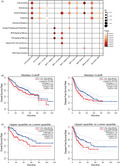

## 1 INTRODUCTION

Liver cancer is one of the most common cancers worldwide with its incidence ranking fifth and mortality ranking fourth among all malignancies in both sexes.^1^ For patients with early‐stage liver cancer, radical treatment of surgery is the first choice, but most liver cancer patients are diagnosed at an advanced stage.^2^ Considering the limitations and poor outcomes of existing treatments, immunotherapy is a promising therapeutic approach; however, only a small percentage of patients benefit from this treatment.^3^ Therefore, continued research into molecular biomarkers and novel therapies is critical for predicting prognosis and determining personalized treatment.

The growing data from research on tumor microenvironment (TME) indicates that tumor immune status plays a critical role in cancer progression and prognosis.^4^ Multiple TME markers, such as tumor‐infiltrating immune cells and immune‐related gene expression profiles, have provided extensive evidence in diverse cancer types that these markers are useful, not only in cancer prognosis but also in cancer immunotherapy.^5^ Long non‐coding RNAs (lncRNAs), a type of non‐coding RNAs that are greater than 200 nucleotides in length, are critical regulators of gene expression and play a crucial role in immune regulation. For instance, the lncRNA KCNQ1 can play an immunosuppressive role, thereby promoting the immune escape of liver cancer.^6^ Recent studies have found that lncRNAs are correlated with immune‐cell infiltration in different cancers, including liver cancer.^7^ Furthermore, several studies have reported that lncRNA profiles can be used to predict the prognosis of liver cancer.^8^ However, there are currently few studies regarding the relationship between the lncRNAs and the TME in liver cancer.

In this study, we analyzed the Cancer Genome Atlas Liver Hepatocellular Carcinoma (TCGA‐LIHC) cohort data and developed a novel immune‐related lncRNA signature based on the lncRNAs that significantly affect immune pathway activity. We then investigated the prognostic value of this signature in the cohort and evaluated the relationship of the lncRNA signature with mutation profiles and the TME to deepen our understanding of the potential applications of lncRNAs in liver cancer.

## 2 MATERIALS AND METHODS

### 2.1 Data sources

We retrieved TCGA genomic and transcriptomic data, including lncRNA data, for a total of 364 patients available in the TCGA‐LIHC cohort. Multiple omics data and full clinical characteristics of the TCGA‐LIHC cohort were downloaded from the Genomic Data Commons Data Portal. In addition, transcriptomic data and patient clinical information of the colon cancer (adenocarcinoma) cohort TCGA‐COAD and stomach cancer (adenocarcinoma) cohort TCGA‐STAD were obtained from TCGA and used for validation.

### 2.2 Immune‐related lncRNAs

Immune‐related lncRNAs were obtained from the ImmLnc database (http://bio‐bigdata.hrbmu.edu.cn/ImmLnc).^9^ The immune‐related lncRNAs were obtained in three steps. First, genome‐wide and lncRNA‐specific gene expression data were collected for the same patient. Then, all coding genes were ranked based on their correlation with each lncRNA expression. Finally, the genes in each immune‐related pathway were mapped to a ranked gene list and the enrichment scores (ES) were calculated based on the Gene Set Enrichment Analysis (GSEA), which was converted to a lncRES score. LncRNA‐pathway pairs with a lncRES score of >0.995 and false discovery rate (FDR) of <0.05 were selected as immune‐related lncRNAs. The immune‐related lncRNAs of liver cancer were downloaded from the ImmLnc database.

### 2.3 Signature development of immune‐related lncRNAs

A total of 2908 immune‐related lncRNAs in liver cancer were obtained, 699 of which were negatively correlated with immune‐related pathways. The lncRNAs were ranked according to the sum of their ES in immune‐related pathways, and the 10 most significantly negatively correlated lncRNAs were selected to build the lncRNA signature. Expression data for the 10 lncRNAs were extracted from the transcriptomic data of the TCGA‐LIHC cohort, log2 transformation performed was on transcripts per million (TPM) values for each lncRNA, and the quantitative values were summed. This ultimately resulted in a lncRNA signature score for each patient. The patients were divided into two groups, the low lncRNA signature group (Lnc_low) and the high lncRNA signature group (Lnc_high), based on the median value of the lncRNA signature score. The relationship between lncRNA signatures and clinical outcomes in the TCGA‐LIHC cohort was then explored. The prognostic value of the lncRNA signature was validated in the TCGA‐COAD and TCGA‐STAD cohorts.

### 2.4 Molecular features

Single nucleotide variant and small insertion‐deletion (INDEL) data were derived from the genomic data of the TCGA‐LIHC cohort. The tumor mutation burden (TMB) and copy number variation (CNV) burden of the cohort were derived from data of a published study.^10^ The top 10 genes with different somatic mutation types in the Lnc_low and Lnc_high groups were displayed in a heatmap based on the frequency of the genes. The difference in gene mutation frequency between the two groups was compared using Fisher's exact test. The genes were then grouped into eight known canonical pathways that included P53, WNT, RTK‐RAS, TGFβ, Hippo, cell cycle, PI3K, and Notch, as previously described.^11^ Samples in which genes of specific pathways contained somatic mutations were designated as having specific pathway alterations. Differences in the cancer pathway alteration frequency between the Lnc_low and Lnc_high groups were compared using Fisher's exact test.

### 2.5 TME

Using genomic and transcriptomic data from the TCGA‐LIHC cohort, we assessed the TME, including cancer immunograms, 28 immune‐cell subsets, five immune checkpoints, nine immune gene signatures, and immune subtypes. In accordance with a previous study, the cancer immunogram was designated using eight axes of the immunogram score (IGS), which reflect the seven steps in the cancer immunity cycle.^12^ The IGS axes included IGS1, T‐cell immunity; IGS2, tumor antigenicity; IGS3, priming and activation; IGS4, trafficking and infiltration; IGS5, recognition of tumor cells; IGS6, inhibitor cells; IGS7, checkpoint expression; and IGS8, inhibitory molecules. Gene set variation analysis (GSVA) was performed using the GSVA R package to assess the value of IGS. The differences in the eight axes of the Lnc_low and Lnc_high groups of the TCGA‐LIHC cohort were displayed as a heatmap based on IGS values. Two groups of immunogram radar figures showed the median values of ranked IGS. Distribution of the 28 immune‐cell subsets and expression of the five immune checkpoint molecules in the two groups were calculated as the geometric mean of log2 gene expression of TPM+1 using GSVA R packages. The gene sets for cytolytic activity, IFN‐γ signature, immunocostimulators, immunoinhibitors, chemokines, T‐cell‐inflamed gene expression profile, and MHC‐class‐I/II signature were defined as previously reported.^13^ Immune gene signatures were measured as the mean value of log2 gene expression of TPM+1. We obtained the immune subtypes of the TCGA‐LIHC cohort using the supplementary data provided by Thorsson et al^10^ and compared the differences in immune types (C1, wound healing; C2, IFN‐γ dominant; C3, inflammatory; C4, lymphocyte depleted; C5, immunologically quiet; C6, TGF‐β‐dominant) between the Lnc‐low and Lnc‐high groups.

### 2.6 Statistical analysis

The data were analyzed using R 3.6.1 and SPSS software version 24.0. Survival analysis was performed using Kaplan‐Meier curves and compared between groups using a log‐rank test. The chi‐squared test or Fisher's exact test was used to analyze the association between various genomic determinants. The student's *t* test was used to analyze the differences between the two groups when the data were normally distributed; otherwise, the Mann‐Whitney *U* test was used. Statistical significance was set at *p* < 0.05.

## 3 RESULTS

### 3.1 Immune‐related lncRNA signature and prognosis in liver cancer

**FIGURE 1:**
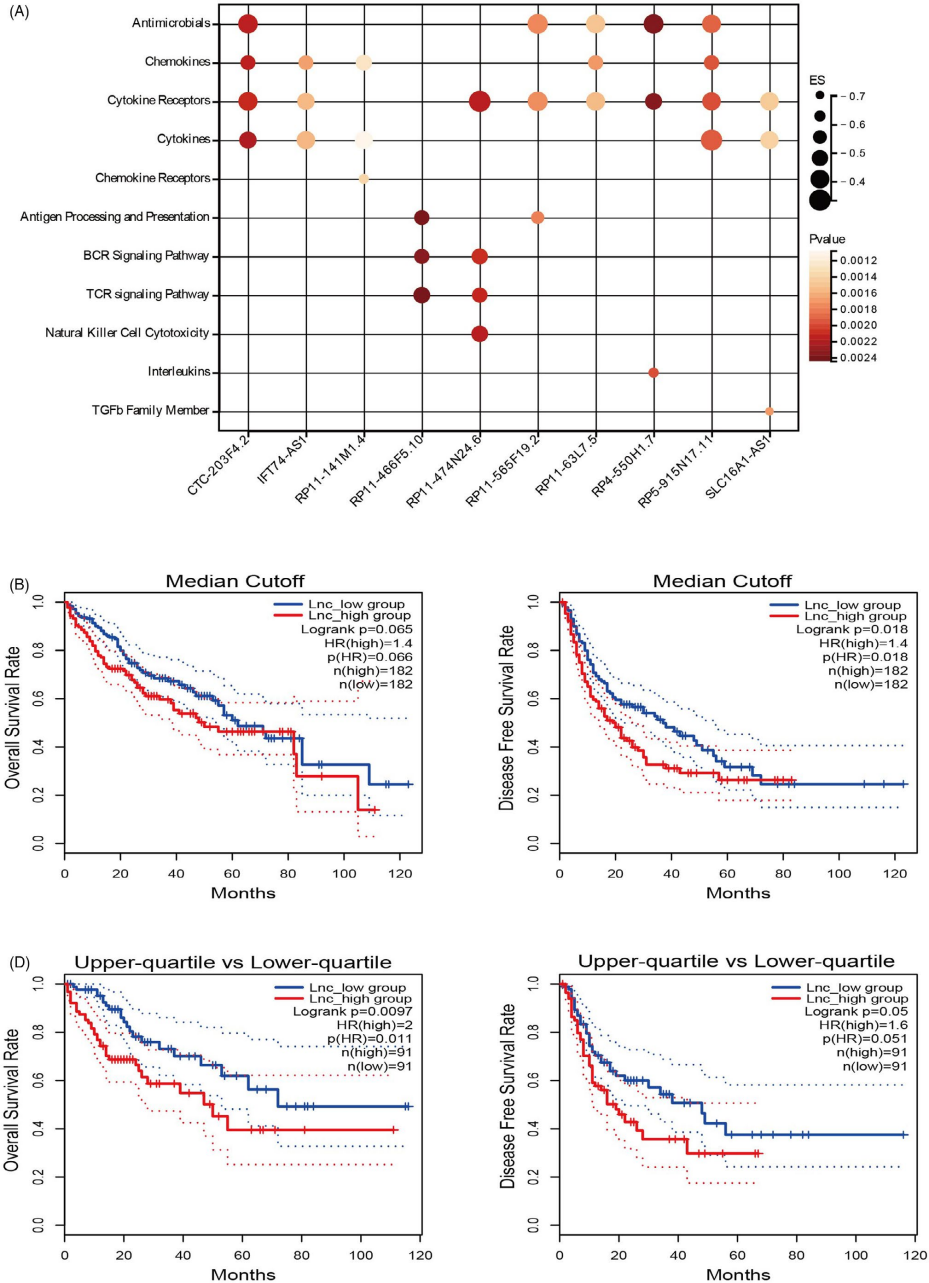
Immune‐related lncRNA signature and prognosis in liver cancer. (A) Characterization of the top 10 inversely related immune lncRNAs in the TCGA‐LIHC cohort. (B) Lower levels of a combined 10 lncRNA signatures associated with improved overall survival (OS) and (C) disease‐free survival (DFS)

The significant lncRNA‐pathway pairs in liver cancer with an absolute lncRES of >0.995 and an FDR of <0.05 were downloaded from the ImmLnc Database. A total of 2908 lncRNAs were significantly correlated with immune‐related pathways, among which 699 were negatively correlated with immune‐related pathways. We ranked these lncRNAs according to the sum of their ES in immune‐related pathways. The 10 most significantly negatively correlated lncRNAs were selected and a lncRNA signature was obtained using logarithmic transformation of their TPM values. The 10 immune negatively related lncRNAs were CTC‐203F4.2, IFT74‐AS1, RP11‐141M1.4, RP11‐466F5.10, RP11‐474N24.6, RP11‐565F19.2, RP11‐63L7.5, RP4‐550H1.7, RP5‐915N17.11, and SLC16A1‐AS1. These lncRNAs were mainly correlated with “cytokine receptors,” “cytokines,” “chemokines,” and “antimicrobials” pathways (Figure 1A).

We then performed a prognostic analysis of TCGA‐LIHC patients stratified by the lncRNA signature based on DFS and OS. A score was obtained for each patient with respect to each of the 10 lncRNAs. First, we used the median of the scores as the cutoff value and divided the patients into two groups, Lnc_high and Lnc_low. Kaplan‐Meier curves with log‐rank analysis showed that the Lnc_high group of patients had a shorter DFS compared with that of the Lnc_low group (HR = 1.4, *p* = 0.018), even though there was no significant difference in OS (HR = 1.4, *p* = 0.066) between the two groups (Figure 1B). Then, setting the quartile as the cutoff value, we found that the top 25% group had shorter OS (HR = 2, *p* = 0.011) compared with that of the bottom 25% group, and DFS showed a tendency for worse prognosis (HR = 1.6, *p* = 0.051) with the *p* value being close to the threshold of significance (Figure 1C).

To verify the prognostic value of the lncRNA signature, we applied the lncRNA signature and determined the OS of colorectal cancer and gastric cancer patients. Setting the quartile as the cutoff value, via the online resource Gene Expression Profiling Interactive Analysis 2 (GEPIEA2), we found that lower levels of the lncRNA signature were associated with improved OS in both colorectal cancer (HR = 3.5, *p* = 0.0022) and gastric cancer (HR = 1.6, *p* = 0.047) in the TCGA data sets (Figure S1).

### 3.2 Relationship of the lncRNA signature with clinicopathological features

**FIGURE 2:**
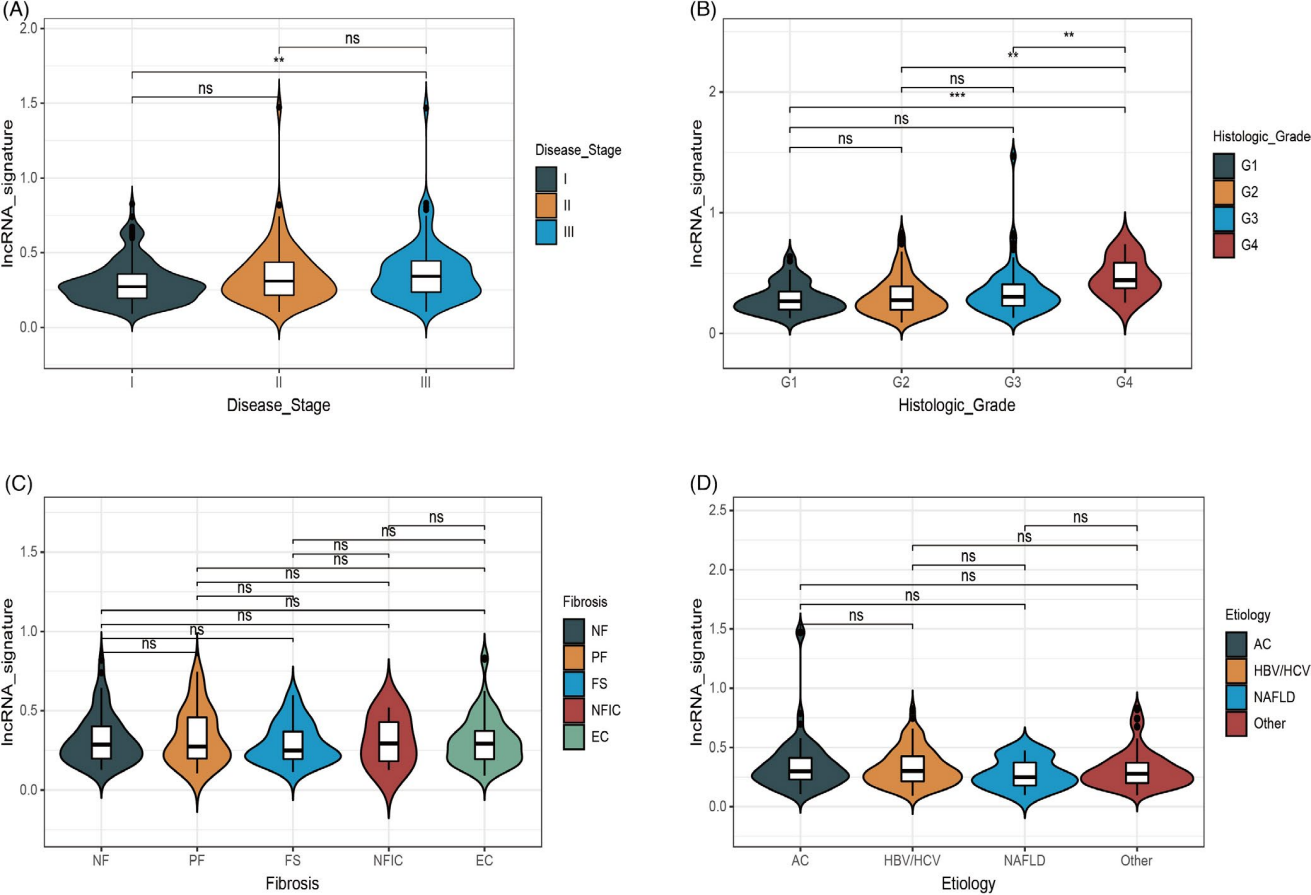
Relationship of the lncRNA signature with clinicopathological features. Correlation between disease stage (A), histologic grade (B), liver fibrosis ishak score category (C), and etiology (D), and the expression level of the lncRNA signature. AC, alcohol consumption; EC, established cirrhosis; FS, fibrous speta; HBV/HCV, hepatitis B/hepatitis C; NAFLD, non‐alcoholic fatty liver disease; NF, no fibrosis; NFIC, nodular formation and incomplete cirrhosis; PF, portal fibrosis

Next, the correlations between clinicopathological features and the lncRNA signature were analyzed. The expression level of the lncRNA signature increased progressively with disease stage from stage I to stage III (Figure 2A) and also with histologic grade from grade 1 to grade 4 (Figure 2B). These results suggested that the 10 lncRNA signature may be involved in the progression of HCC development. We also explored the asscociation between etiology or liver fibrosis ishak score category and the lncRNA signature separately. However, we did not observe any significant difference among different subgroups (Figure 2C,D).

### 3.3 Distinct mutational profiles in the low and high lncRNA signature subgroups

**FIGURE 3:**
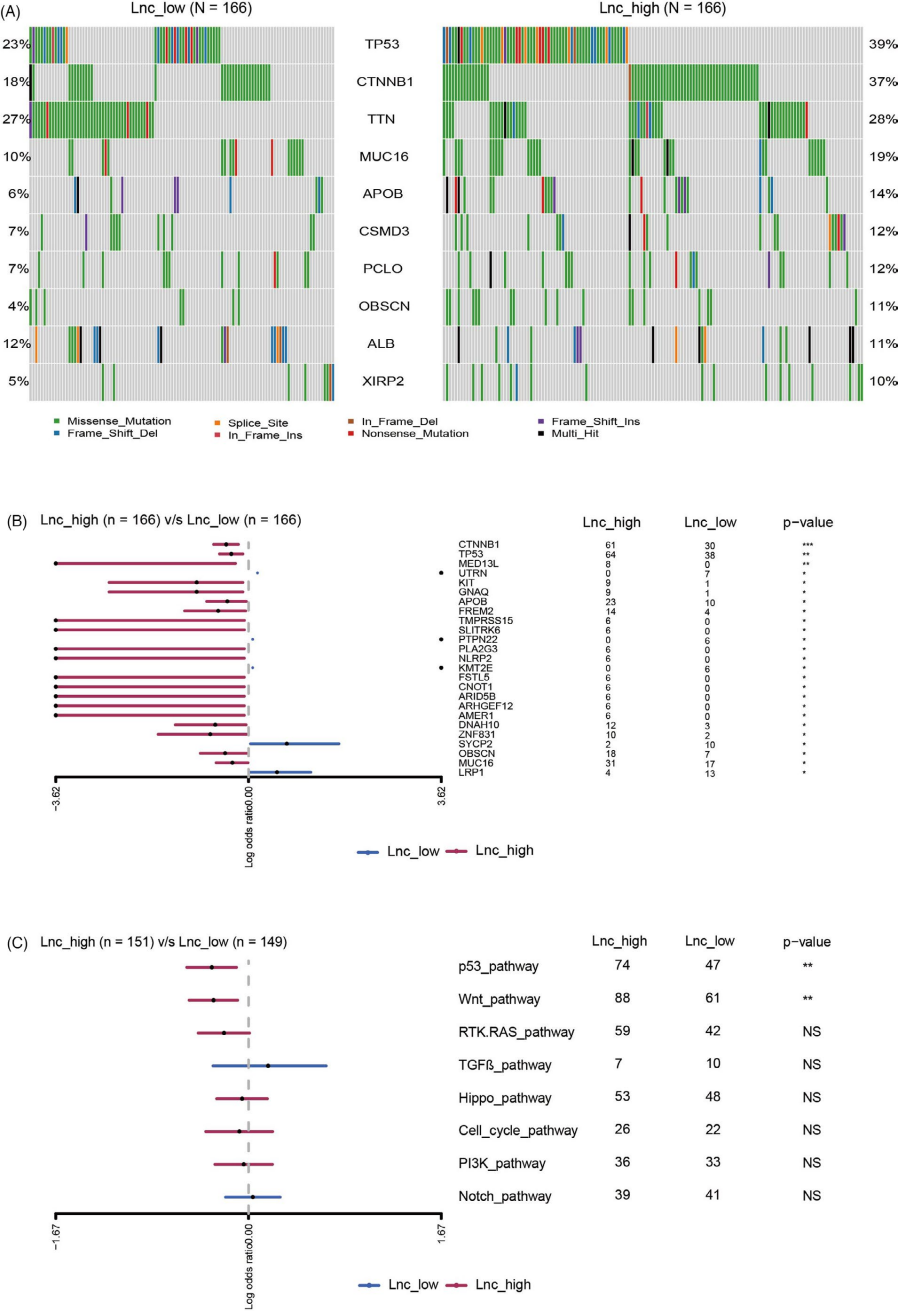
Distinct mutational profiles in low and high lncRNA signature subgroups. (A) Comparison of the top 10 frequently mutated genes between the Lnc_low group and Lnc_high group. (B) Comparison of the genes with significantly different mutation frequency between the Lnc_low and Lnc_high groups. (C) Comparison of eight canonical pathways between the Lnc_low and Lnc_high groups

To investigate the relationship between the lncRNA signature and mutational profiles, we analyzed the liver cancer genomic data of the TCGA‐LIHC cohort. As shown in Figure 3A, the frequencies of the top 10 frequently mutated genes in the Lnc_high group differed from those in the Lnc_low group. Comparison of the genes with significantly different mutation frequencies between the two groups revealed that the number of mutations on 20 genes was higher in the Lnc_high group, especially for *CTNNB1* (61 vs 30, *p* < 0.001), *TP53* (64 vs 38, *p* < 0.01), and *MED13L* (8 vs 0, *p* < 0.01; Figure 3B). Meanwhile, only five genes in the Lnc_low group had a higher number of mutations than that in the Lnc_high group. We also found that the number of mutations on genes involved in the p53 and WNT pathways was higher in the Lnc_high group compared with that in the Lnc_low group (two‐sided Fisher's exact test, *p* < 0.01; Figure 3C). The other signaling pathways evaluated, including the RTK‐RAS, TGFβ, Hippo, cell cycle, PI3K, and Notch pathways, were not significantly different between the two lncRNA signature groups (two‐sided Fisher's exact test, *p* > 0.05; Figure 3C). Interestingly, we did not identify any significant difference in TMB and CNV burden between the lncRNA signature high and low subgroups (Figure S2).

### 3.4 Higher levels of the lncRNA signature correlated with an immune‐excluded TME

**FIGURE 4:**
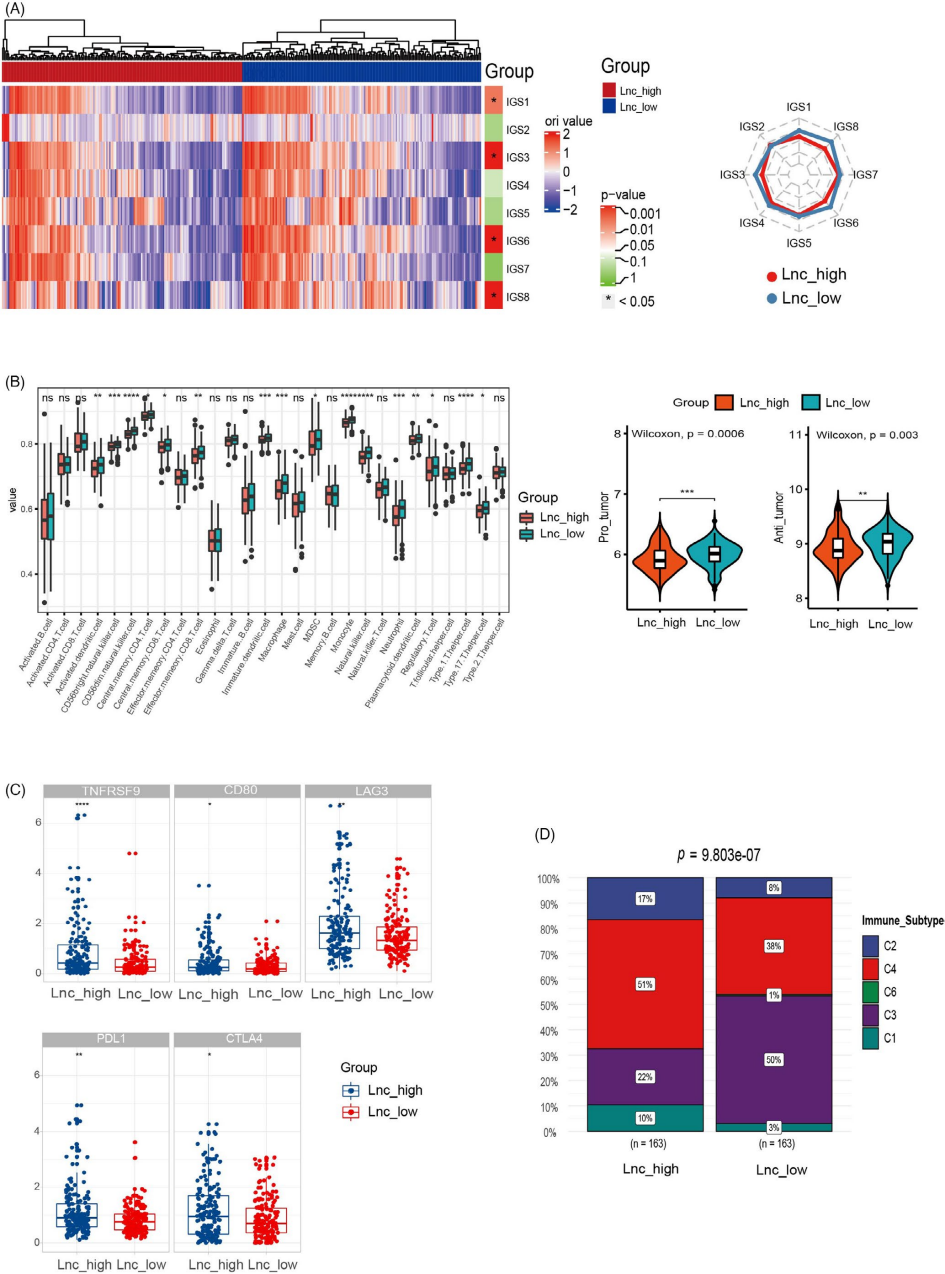
Higher levels of lncRNA signature correlate with an immune‐excluded tumor microenvironment. (A) Comparison of the eight immune cycle axes of the cancer immunogram between the two groups. (B) Comparison of the 28 immune‐cell subsets between the Lnc_low group and Lnc_high group. (C) Comparison of five immune checkpoint molecules between the Lnc_low and Lnc_high groups. (D) Distribution of immune subtypes between the Lnc_low and Lnc_high groups

We analyzed the transcriptomic data of the TCGA‐LIHC data set to explore the association of the lncRNA signature with the immune microenvironment. First, to examine the immunological features between the high lncRNA signature group and low lncRNA signature group, we adopted a cancer immunogram that visually illustrates the state of the cancer immunity cycle. As shown in Figure 4A, four axes of the IGS, including IGS1, IGS3, IGS6, and IGS8, were significantly higher in the Lnc‐low group compared with those in the Lnc‐high group. The other four axes were not significantly different between the two groups. Next, we used single‐sample GSEA (ssGSEA) to evaluate the relative abundance of 28 immune‐cell subsets that infiltrated the tumors. Six of 15 adaptive immune‐cell subsets and 10 of 13 innate immune‐cell subsets infiltrated the tumors of patients in the Lnc_low group at significantly higher levels compared with those in the Lnc_high group (Figure 4B). When the immune‐cell subsets were further classified into protumor and antitumor groups, we found that both the protumor‐related and antitumor‐related immune‐cell subsets infiltrated tumors in the Lnc_low group at significantly higher levels compared with those in the Lnc_high group (Figure 4B). In addition, we observed a higher level of immunoinhibitory immune signature in the Lnc‐high group (Figure S3). We also analyzed the expression of immune checkpoint molecules between the two lncRNA groups and found that the expression levels of *TNFRSF9*, *CD80*, *LAG3*, *PD*‐*L1*, and *CTLA4* were significantly higher in the Lnc_high group (Figure 4C). Finally, we obtained the immune subtype data of the TCGA‐LIHC cohort analyzed by Thorsson et al.^10^ and found a significant difference between the Lnc_high group and the Lnc_low group (*p* < 0.01). The proportion of the C4 immune subtype was the highest in the Lnc_high group (51%), while the proportion of C3 immune subtype was the highest in the Lnc_low group (50%). The proportion of each immune subtype in the samples belonging to the two groups is shown in Figure 4D.

## 4 DISCUSSION

In recent years, significant progress has been made in the treatment of liver cancer, especially with the breakthrough of immunotherapy, which has allowed liver cancer intervention to enter a new era. Many advances have shown that the TME plays an important role in tumor development and is closely related to immunotherapy.^14^, ^15^ LncRNAs are emerging as critical regulators of the genome network and play critical roles in the development and activation of immune cells and immune regulation.^16^ Here, we constructed a novel signature based on immune‐related lncRNAs in patients with liver cancer that delineates an immune‐excluded subtype of liver cancer with unfavorable clinical outcomes.

Immune‐related lncRNA signatures have been explored in non‐small cell lung cancer, breast cancer, and liver cancer. Meanwhile, co‐expression analyses of lncRNAs and immune genes or immune cells have mainly been used to generate immune‐related lncRNAs, showing that immune‐related lncRNA signatures can be used to effectively predict the prognosis of patients.^17^, ^18^, ^19^ Here, the immune‐related lncRNAs were obtained from the ImmLnc Database, which focuses on the lncRNAs that affect immune pathway activity. We constructed a lncRNA signature based on the top 10 inversely related immune‐lncRNAs. The signature was shown to be correlated with poor prognosis (Figure 1), as well as progressed disease stage and histologic grade (Figure 2) in the TCGA‐LIHC cohort data set. The lncRNA expression was mainly correlated with the “cytokine receptors,” “cytokines,” “chemokines,” and “antimicrobials” pathways, suggesting lncRNAs that regulate the immune pathway may play a crucial role in liver cancer progression and prognosis. Intriguingly, among the 10 lncRNAs, SLC16A1‐AS1 have been reported correlated with poor prognosis in several cancers including liver cancer,^20^, ^21^ while others have not been reported yet. We validated the prognostic value of the lncRNA signature in colorectal and gastric cancer and found that the 10 lncRNA signatures were useful for predicting the prognosis of these cancers. This indicates that the lncRNA signature we generated from liver cancer data may have a universal function in gastrointestinal cancer.

Next‐generation sequencing has provided a wealth of information about genomic characteristics in a variety of cancers. For instance, *TP53* and *CTNNB1* are two of the most important genes in liver cancer with variants occurring at very high frequencies. *TP53* mutations are strongly related to the immune microenvironment, resulting in the downregulation of the immune response in hepatocellular carcinoma.^22^ *CTNNB1* mutations are thought to characterize the immune‐excluded class of hepatocellular carcinoma and may be a biomarker for predicting resistance to immune checkpoint inhibitors.^23^ Here, we found that the Lnc_high group had a significantly higher mutation frequency for both *TP53* and *CTNNB1* compared with that in the Lnc_low group. We also found that the number of mutations in the p53 and WNT pathways was higher in the Lnc‐high group compared with that in the Lnc‐low group. These findings suggest that high lncRNA signature levels may be associated with a cold immune microenvironment. TMB and CNV burden are well‐known genomic signature related to immunological infiltrations, while we did not found any difference in TMB and CNV burden between the Lnc_high group and Lnc_low group.^24^, ^25^ This indicated that the associations between the lncRNA signature and immune microenvironment might be independent of TMB or CNV burden.

In recent years, with the development of checkpoint inhibitor‐based immunotherapies, several immune checkpoint inhibitors have been applied in the clinical practice against liver cancer, including pembrolizumab, nivolumab, and atezolizumab, providing significant survival benefits to many patients. However, similar to non‐small cell lung cancer and other cancers, only a small subset of patients benefit from immune checkpoint blockade therapy. This is even true for combination immunotherapy, such as atezolizumab combined with bevacizumab, with less than one‐third of patients in the Imbrave150 clinical trial showing a response.^26^ Currently, commonly used biomarkers, including PD‐L1 expression and tumor mutation burden, may play a role in patient selection for immunotherapy in some cancers, such as lung cancer, but they have very limited significance for liver cancer. The TME is increasingly recognized as a promising biomarker associated with immunotherapy responses.^5^ Here, we found four axes of the IGS, IGS1, IGS3, IGS6, and IGS8, that were significantly higher in the Lnc_low group compared with those in the Lnc_high group. We also found that our immune‐related lncRNA signature correlated with immune‐cell infiltration. These findings suggest that immune‐related lncRNA signatures are significantly correlated with the TME and may be integrated into a comprehensive biomarker system for immunotherapy.

In addition, we observed higher immunoinhibitor immune signature levels and expression levels of inhibitory checkpoints in the Lnc_high group compared with that in the Lnc_low group, which indicated that the tumors with Lnc_high signature were more likely to exhibit an immunosuppressive state and tended to escape immune cells within the TME. Recent studies have recognized that the overexpression of immune checkpoints, such as PD‐L1, CTLA4, and LAG3, is associated with poor prognosis in liver cancer,^27^, ^28^ which is consistent with our result of the Lnc_high group having poor DFS and OS. Analyzing immune subtypes is another method to identify the composition of TME. The proportion of the C4 subtype was the highest immune subtype in the Lnc_high group, reaching 51%. Meanwhile, the proportion of the C3 subtype was the highest immune subtype in the Lnc_low group, reaching 50%. This indicates that tumors of the Lnc_high group may represent an immune‐excluded subtype of liver cancer.

In conclusion, we constructed a novel immune‐related lncRNA signature based on lncRNAs that affect immune pathway activity and determined that this lncRNA signature is correlated with the progression and prognosis of liver cancer. The immune‐related lncRNA signature demonstrates a significant relationship with tumor mutations and the TME and may be integrated into a comprehensive biomarker system for immunotherapy. However, this immune‐related lncRNA signature needs to be applied to patients in a clinical setting to further verify its role in the prognosis of liver cancer and its ability to predict the efficacy of immunotherapy.

## CONFLICT OF INTEREST

The author(s) declared no potential conflict of interest with respect to the research, authorship, and/or publication of this article.

## AUTHOR CONTRIBUTIONS

All listed authors participated in the study design, analysis, and interpretation of the data, drafted or revised the article, have agreed on the journal submitted currently, and given approval for the version to be published.

## Supporting information

Fig S1

Fig S2

Fig S3

Supplementary Material

## Data Availability

The data can be obtained from TCGA database (https://portal.gdc.cancer.gov/).
