# Supplementary material for: Immune‐related lncRNA signature delineates an immune‐excluded subtype of liver cancer with unfavorable clinical outcomes
Source: J Clin Lab Anal. 2022 Jan 18;36(3):e24244. doi: 10.1002/jcla.24244 (PMC8906039; doi:10.1002/jcla.24244)
Supplement: Supplementary file 4 — Supplementary Material [file JCLA-36-e24244-s003.docx]

**Supplementary figure legends**

**Supplementary Figure S1. Prognostic value of the lncRNA signature in colorectal cancer (COAD) and gastric cancer (STAD).** Lower levels of the ten lncRNA signatures are associated with improved overall survival (OS) in colorectal cancer (A) and gastric cancer (B) in the TCGA data set, as determined via the online resource GEPIEA2.

**Supplementary Figure S2. Comparison of the TMB(A) and CNV burden(B) between the Lnc_high group and Lnc_low group.**

**Supplementary Figure S3. Higher lncRNA signature levels correlate with increased expression levels of immune-checkpoint molecules.**
